# Neighbourhood migrant density and outcomes in hospitalised patients with cancer before and during the COVID-19 pandemic in Sweden: a register-based retrospective cohort study

**DOI:** 10.1136/bmjopen-2025-113681

**Published:** 2026-05-18

**Authors:** Yu Par Khin, Takeo Fujiwara, Mikael Rostila, Daniel Nigusse Tollosa, Alexander Miething

**Affiliations:** 1 Department of Public Health, Institute of Science Tokyo Graduate School of Medical and Dental Sciences, Bunkyo, Japan; 2 Department of Public Health Sciences, Stockholm University, Stockholm, Sweden

**Keywords:** COVID-19, Cancer, Hospitalization, PUBLIC HEALTH, Cancer Survivors, EPIDEMIOLOGY

## Abstract

**Abstract:**

**Objectives:**

Neighbourhood migrant density is increasingly recognised as a social determinant of health. However, its association with hospitalised patients with cancer outcomes, such as mortality and readmission rates, remains understudied. This study examined whether neighbourhood migrant density influenced these outcomes and whether these associations varied before and during the COVID-19 pandemic.

**Design:**

Retrospective cohort study.

**Setting:**

Swedish national registers.

**Participants:**

Hospitalised patients with cancer (ICD code C00–C97) from 2014 to 2019 (before the pandemic) and 2020–2021 (during the pandemic).

**Outcomes:**

90-day mortality and readmission rates. Independent variables were neighbourhood migrant density—categorised as total, Western and non-Western migrants (as a proportion of the total area population).

**Results:**

We identified 243 357 hospitalised patients with cancer before the pandemic and 112 935 during the pandemic. Swedish-born individuals and Western migrants residing in high migrant density neighbourhoods had higher rates of 90-day mortality (incidence rate ratio, IRR: 1.15, 95% CI 95% CI 1.12 to 1.19 and IRR: 1.09, 95% CI 1.00 to 1.18) and readmission (IRR: 1.16, 95% CI 1.13 to 1.19 and IRR: 1.14, 95% CI 1.07 to 1.22). During the pandemic, 90-day mortality rates significantly increased among Western migrants and 90-day readmission rates increased for all patients from high migrant density neighbourhoods.

**Conclusions:**

High neighbourhoods migrant density was associated with increased 90-day mortality and readmission among Swedish-born individuals and Western migrants before the pandemic. These outcomes were exacerbated during the pandemic, particularly among migrants. Cancer care for residents in neighbourhoods with high migrant density needs to be improved, especially during public health crises.

**Strengths and limitations of this study**

- Uses nationwide register data for the comprehensive analysis.
- Lacks distinction between different cancer types and stages.
- Includes only hospitalised patients with cancer, excluding those who had delayed diagnosis or never reached hospitalisation.

## Introduction

 Like many high-income countries, Sweden has an increasing ageing population and a high burden of cancer, which was the second-leading cause of death in 2021.^1^ Migrants in Sweden generally exhibit the ‘healthy migrant effect’ characterised by low overall cancer incidence and cancer-related mortality.^2^,^5^ Previous studies have shown that being born outside of Sweden has no significant association with duration and frequency of cancer hospitalisation^6^ and migrants also exhibited a higher chance of survival after treatment.^7^ However, Western migrants have also been reported to experience higher mortality rates from tobacco-related cancers and non-Western migrants from infection-related cancers.^3 4 8^

Currently, foreign-born population accounts for 20% of the population in Sweden, which is the largest among the Nordic countries in 2022.^9^ Migrants in Sweden can be categorised by distinct waves of migration: labour migration from Western countries dominated until the 1950s–1960s, followed by refugee and asylum-seeking migration from non-Western nations after the 1970s.^10^ Newcomer migrants from non-Western countries with limited socioeconomic resources are often concentrated in specific suburban neighbourhoods due to limited housing options.^11^ These differ significantly from neighbourhoods with predominantly Swedish-born populations in terms of socioeconomic conditions and social structure, denoted by high unemployment and crime rates.^12^ Typically, these less advantaged areas are the only places where affordable housing is available to both native and migrant households with limited financial resources.^13^ For patients with cancer, though timely and effective care is ensured once treatment begins,^14^ migrant-dense neighbourhoods tend to have fewer primary healthcare practices,^15^ which serve as the primary entry point to the healthcare services and are crucial for early detection of cancer. Limited availability of health-promoting services, together with broader adverse social determinants, may affect healthcare access for all residents of these neighbourhoods.^16^

The outbreak of the COVID-19 pandemic exacerbated existing healthcare disparities in migrant-dense neighbourhoods.^17^ Those residing in neighbourhoods with high migrant density had higher COVID-19 related mortality^17^ and hospitalisation rates,^18^ though further research is needed to clarify the underlying mechanism. These challenges were more pronounced for patients with cancer, who faced a higher risk of COVID-19 infection and related complications.^19^ This higher infection risk hindered their access to healthcare, delaying their cancer treatment. Hospitalisation is often inevitable for cancer treatment, and patients with cancer residing in migrant-dense neighbourhoods—who had already been vulnerable—became even more so during the COVID-19 pandemic.

However, limited research has been conducted on the outcomes of hospitalised patients with cancer residing in migrant-dense neighbourhoods. The 90-day mortality and readmission are commonly used indicators for the quality of hospitalised cancer care, which is often compromised in migrant-dense neighbourhoods due to the limited access to care.^20^ Therefore, this study examines the associations between neighbourhood migrant density and 90-day mortality and readmission rates of hospitalised patients with cancer before the COVID-19 pandemic, and how these associations were modified during the pandemic.

## Methods

### Study design and population

We used multiple longitudinal national registers linked with unique pseudonymised personal identifiers, covering the total population of Sweden. The linked datasets include the total population register, the Longitudinal Integration Database for Health Insurance and Labour Market Studies (LISA) and the National Outpatient and Inpatient Register. The total population register covers the entire population of Sweden from 1968 onwards.^21^ The LISA contains information on all Swedish residents aged 16 years and above.^22^ Both the national outpatient and inpatient registers have nationwide coverage from both public and private healthcare providers.^23^ Therefore, using these nationwide registers allows complete coverage of the Swedish resident population.

We included individuals who were admitted to hospitals with a cancer diagnosis. The study period was from 2014 to 2019 for ‘before the pandemic’ period and from 2020 to 2021 for ‘during the pandemic’ period. Individuals were included from 18 years old, or from the time they immigrated to Sweden after 18 years old. Individuals who had been hospitalised for cancer before the pandemic and survived were eligible to be included in the study during the pandemic period. Follow-up ended at the time of death or emigration out of Sweden. There were no missing values in the variables of the analytic dataset.

### Outcome

We used hospital inpatient data to identify individuals with a history of hospitalisation for any type of malignant tumour (ICD-10 codes C00–C97). The 90-day mortality was coded as a binary variable (0/1) based on whether the patient died from any cause within 90 days of the admission date. The 90-day readmission was defined as a readmission with a cancer diagnosis within 90 days of the previous discharge date. Individuals who were readmitted within 90 days and subsequently died within 90 days were eligible for both outcome measures.

### Exposure

The migrant population was classified into all migrants, Western and non-Western migrants to reflect differences in socioeconomic condition and health outcomes between migrant groups, following previous studies.^24 25^ Western migrants were defined as individuals originating from European countries, the USA, Canada, Australia or New Zealand, while non-Western migrants were defined as those from all other regions.

The main exposure, migrant density, a compositional measure of migrant residential segregation,^26^ was calculated by dividing the migrant population in a specific geographic area by the total population of that area. We then stratified this measure into Western and non-Western migrant density, as supplementary analyses, to examine whether associations differed across migrant groups residing in neighbourhoods with similar or different migrant background compositions. We used DeSO (Demografiska statistikområden) units as the geographic boundaries.^27^ Each DeSO unit is identified by a nine-digit code and divides Sweden into 5984 areas, each comprising between 700 and 2700 inhabitants. Migrant density of each neighbourhood was included as the first year of observation, as we assumed that neighbourhood characteristics are unlikely to change over a short time span, and that individuals are likely to relocate to neighbourhoods of similar migrant density. Migrant density was further categorised into three quantiles—low, medium and high.

### Covariates

Individual-level covariates included age (categorised into 5-year intervals), sex (male or female), education level (primary, secondary or post-secondary), civil status (with or without a partner), the comorbidity index measured in the first year of observation and the mean disposable income during the study period. The National Cancer Institute Comorbidity Index 2021,^28^ developed specifically for patients with cancer, was calculated using the national outpatient dataset to comprehensively cover possible comorbidities. The comorbidity index was categorised as 0, 1, 2 or 3 and above. Since disposable income, covering all sources of income, can significantly fluctuate over time, we calculated the mean disposable income across each study period and categorised it into quartiles. The income information was derived from the LISA database, which collects income data from national registers, including those of the Swedish tax authorities and social welfare services.^22^ Area-level deprivation for each DeSO unit was included as a time-fixed contextual covariate, measured at the first year of observation. The deprivation index combined information on the proportion of adult residents within each DeSO who were living in relative poverty (defined as having less than 60% of the mean disposable income), not in employment, receiving social assistance benefits and having completed only lower secondary education or less.^26^ The index was divided into ten deciles and ranged from the least deprived (decile 1) to the most deprived (decile 10).

### Analysis

To estimate the associations between neighbourhood migrant density and the outcomes separately for before and during the pandemic, we used multilevel Poisson regression with robust variance, which is appropriate for common outcomes (more than 10%). A multilevel model with a random intercept was specified, with individuals nested within DeSO units. Model fit in the multilevel analyses was assessed using the intraclass correlation coefficient (ICC), which ranges up to 0.4. Despite small ICC values, we maintained multilevel models to account for the hierarchical structure of data, as ignoring clustering may result in underestimated SEs, even with low between-group variance.^29^ The logarithm of follow-up person-years was included as an offset. Analyses were stratified into three population groups: Swedish-born, Western migrants and non-Western migrants. Incidence rate ratios (IRRs) were calculated to estimate the 90-day mortality and 90-day readmission. Model 1 was adjusted for age, sex, disposable income, education level, civil status and comorbidity index, while model 2 was additionally adjusted for the area-level deprivation index. To confirm the differences in associations between neighbourhood migrant density and outcomes by population groups, we conducted an interaction analysis. To account for the age distributions between Swedish-born and migrant populations, we conducted age-stratified analysis, categorising participants into age groups younger than 65 and 65 years and older for all outcomes as a sensitivity analysis. Statistical significance was set at p<0.05.

## Results

Table 1 presents the descriptive characteristics of participants before the pandemic and stratified as Swedish-born, Western migrants and non-Western migrants. During this period, 243 357 patients were hospitalised for cancer. Of these, 17.6% (n=42 894) died within 90 days of admission and 23.4% (n=56 834) were readmitted within 90 days of discharge.

**Table 1:** Characteristics of the participants before the pandemic (2014–2019)

	Total	Swedish-born	Western migrants	Non-Western migrants
Number (%)	243.357	210 233 (86.4%)	23 984 (9.9%)	9140 (3.8%)
Person-years	1 260 493	1 088 662	123 156	48 675
Age in 2014 in years (mean, SD)	67.0 (13.3)	67.5 (13.0)	66.8 (12.7)	54.2 (13.8)
Sex				
Men	121 033 (49.7%)	105 714 (50.3%)	11 056 (46.1%)	4263 (46.6%)
Women	122 324 (50.3%)	104 519 (49.7%)	12 928 (53.9%)	4877 (53.4%)
Average disposable income between 2014 and 2019				
First quartile	90 116 (37.0%)	75 011 (35.7%)	11 115 (46.3%)	3990 (43.7%)
Second quartile	66 715 (27.4%)	58 224 (27.7%)	6349 (26.5%)	2142 (23.4%)
Third quartile	46 729 (19.2%)	41 116 (19.6%)	3893 (16.2%)	1720 (18.8%)
Fourth quartile	39 797 (16.4%)	35 882 (17.1%)	2627 (11.0%)	1288 (14.1%)
Education status in 2014				
Primary	74 025 (30.4%)	63 820 (30.4%)	7293 (30.4%)	2912 (31.9%)
Secondary	112 028 (46.0%)	97 442 (46.4%)	11 110 (46.3%)	3476 (38.0%)
Tertiary	57 304 (23.6%)	48 971 (23.3%)	5581 (23.3%)	2752 (30.1%)
Civil status in 2014				
With partner	124 957 (51.4%)	107 837 (51.3%)	11 747 (49.0%)	5373 (58.8%)
Without partner	118 400 (48.7%)	102 396 (48.7%)	12 237 (51.0%)	3767 (41.2%)
NCI Comorbidity Index in 2014				
0	184 673 (75.9%)	159 879 (76.1%)	17 682 (73.7%)	7112 (77.8%)
1	36 893 (15.2%)	31 955 (15.2%)	3858 (16.1%)	1080 (11.8%)
2	14 173 (5.8%)	12 118 (5.8%)	1521 (6.3%)	534 (5.8%)
3+	7618 (3.1%)	6281 (3.0%)	923 (3.9%)	414 (4.5%)
90-day mortality (yes)	42 894 (17.6%)	37 097 (17.7%)	4561 (19.0%)	1236 (13.5%)
90-day readmission (yes)	56 834 (23.4%)	48 329 (23.0%)	6186 (25.8%)	2319 (25.4%)

NCI, National Cancer Institute.

Table 2 shows the descriptive characteristics of participants during the pandemic, when 112 935 patients with cancer were admitted. Among them, 13.9% (n=15 735) died within 90 days of admission and 20.6% (n=23 221) were readmitted within 90 days of discharge. Online supplemental table 1 presents data on neighbourhood migrant density and deprivation index for the years 2014 and 2020. In both periods, non-Western migrants were more likely to reside in deprived neighbourhoods.

**Table 2:** Characteristics of the participants during the pandemic (2020–2021)

	Total	Swedish-born	Western migrants	Non-Western migrants
Number (%)	112.935	96 051 (85.1%)	11 271 (10.0%)	5613 (5.0%)
Person-years	243.346	207.129	24.368	11.849
Age in 2020 in years (mean, SD)	69.8 (13.2)	69.5 (12.9)	68.6 (12.9)	57.2 (13.7)
Sex				
Men	56 870 (50.4%)	48 924 (50.9%)	5236 (46.5%)	2710 (48.3%)
Women	56 065 (49.6%)	47 127 (49.1%)	6035 (53.5%)	2903 (51.7%)
Average disposable income between 2020 and 2021				
First quartile	36 563 (32.4%)	29 675 (30.9%)	4503 (40.0%)	2385 (42.5%)
Second quartile	32 000 (28.3%)	27 498 (28.6%)	3155 (28.0%)	1347 (24.00%)
Third quartile	22 634 (20.0%)	19 564 (20.4%)	2002 (17.8%)	1068 (19.0%)
Fourth quartile	21 738 (19.3%)	19 314 (20.1%)	1611 (14.3%)	813 (14.5%)
Education status in 2020				
Primary	29 107 (25.8%)	24 505 (25.5%)	2888 (25.6%)	1714 (30.5%)
Secondary	54 544 (48.3%)	47 062 (49.0%)	5328 (47.3%)	2154 (38.4%)
Tertiary	29 284 (25.9%)	24 484 (25.5%)	3055 (27.1%)	1745 (31.1%)
Civil status in 2020				
With partner	56 850 (50.3%)	48 209 (50.2%)	5441 (48.3%)	3200 (57.0%)
Without partner	56 085 (49.7%)	47 842 (49.8%)	5830 (51.7%)	2413 (43.0%)
NCI Comorbidity Index in 2020				
0	85 725 (75.9%)	72 935 (75.9%)	8404 (74.6%)	4386 (78.1%)
1	17 146 (15.2%)	14 665 (15.3%)	1795 (15.9%)	686 (12.2%)
2	6640 (5.9%)	5654 (5.9%)	670 (5.9%)	316 (5.6%)
3+	3424 (3.0%)	2797 (2.9%)	402 (3.6%)	225 (4.0%)
90-day mortality (yes)	15 735 (13.9%)	13 413 (14.0%)	1717 (15.2%)	605 (10.8%)
90-day readmission (yes)	23 221 (20.6%)	19 300 (20.1%)	2529 (22.4%)	1392 (24.8%)

NCI, National Cancer Institute.

Table 3 shows the associations between neighbourhood migrant density and the 90-day mortality following cancer hospitalisation, before and during the pandemic, with p values for the interactions between migrant density and population groups. Before the pandemic, Swedish-born individuals living in neighbourhoods with high migrant density were at 1.15 times higher rate (95% CI 1.12 to 1.19) of the 90-day mortality in the fully adjusted model, compared with those living in neighbourhoods with low migrant density. During the pandemic, the incidence rate of 90-day mortality slightly decreased to 1.06 (95% CI 1.01 to 1.12) among the Swedish-born individuals. Before the pandemic, Western migrants living in neighbourhoods with high overall migrant density had 1.09 times (95% CI 1.00 to 1.18) higher rate of 90-day mortality, compared with those living in neighbourhoods with low migrant density. This incidence rate increased to 1.25 (95% CI 1.09 to 1.43) during the pandemic. Associations between neighbourhood migrant density and the 90-day mortality among non-Western migrants were not statistically significant, both before and during the pandemic. However, p for interaction for these migrants was significant (p=0.02). The associations of Western and non-Western migrant density for each population are described in online supplemental table 2.

**Table 3:** Association between neighbourhood migrant density and the 90-day mortality among hospitalised patients with cancer, before the pandemic and during the pandemic

	Before the pandemic (2014–2019)			During the pandemic (2020–2021)		
	Model 1	Model 2	P for interaction	Model 1	Model 2	P for interaction
For Swedish-born						
Low	Ref	Ref	Ref	Ref	Ref	Ref
Medium	1.04 (1.02 to 1.07)*	1.04 (1.01 to 1.07)*	ref	1.03 (0.99 to 1.08)	1.03 (0.99 to 1.07)	Ref
High	1.15 (1.12 to 1.19)*	1.15 (1.12 to 1.19)*	ref	1.09 (1.05 to 1.14)*	1.06 (1.01 to 1.12)*	Ref
For Western migrants						
Low	Ref	Ref	Ref	Ref	Ref	Ref
Medium	0.98 (0.91 to 1.06)	0.98 (0.91 to 1.07)	0.18	1.11 (0.97 to 1.26)	1.12 (0.98 to 1.28)	0.37
High	1.07 (1.00 to 1.16)	1.09 (1.00 to 1.18)*	0.16	1.16 (1.02 to 1.31)*	1.25 (1.09 to 1.43)*	0.88
For non-Western migrants						
Low	Ref	Ref	Ref	Ref	Ref	Ref
Medium	0.89 (0.70 to 1.12)	0.86 (0.68 to 1.09)	0.07	1.05 (0.76 to 1.46)	1.07 (0.77 to 1.49)	0.61
High	0.91 (0.75 to 1.12)	0.86 (0.70 to 1.07)	0.02*	1.07 (0.79 to 1.44)	0.88 (0.63 to 1.23)	0.82

Model 1: adjusted for age, sex, disposable income, education, civil status and comorbidity index.

Model 2: model 1+area level deprivation index.

Neighbourhood migrant density was calculated by dividing the population of each group in a specific geographic area by the total population of that area.

* P value <0.05.

**Figure 1:**
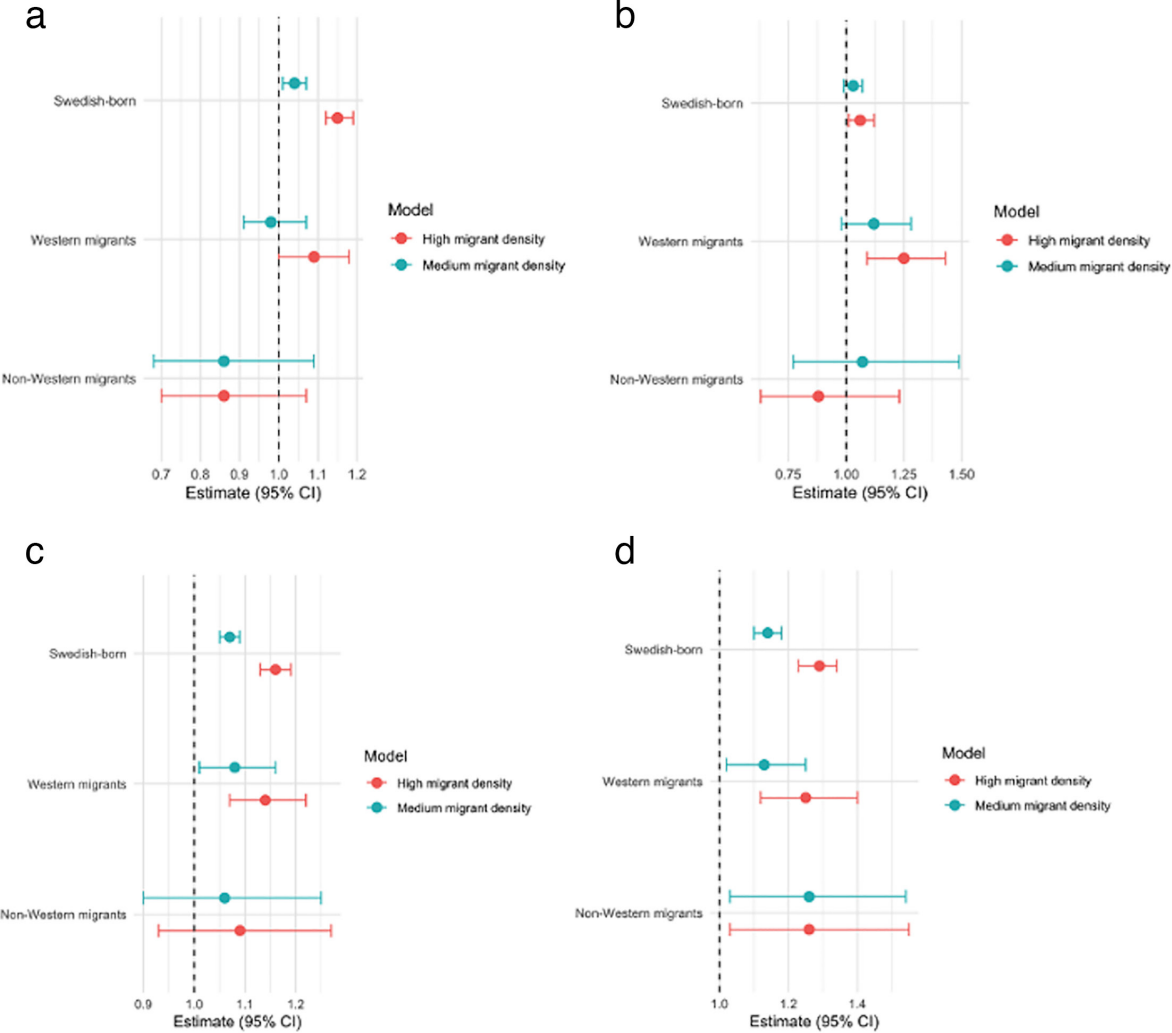
Associations between neighbourhood migrant density and 90-day mortality and 90-day readmission. (a) 90-day mortality (before the pandemic). (b) 90-day mortality (during the pandemic). (c) 90-day readmission (before the pandemic). (d) 90-day readmission (during the pandemic).

Table 4 presents the associations between neighbourhood migrant density and the 90-day readmission following cancer hospitalisation. Before the pandemic, Swedish-born individuals living in neighbourhoods with high migrant density had 1.16 times (95% CI 1.13 to 1.19) higher rate of 90-day readmission compared with those in neighbourhoods with low migrant density. Western migrants exhibited similar associations with the Swedish-born. No significant associations were found for the non-Western migrants. During the pandemic, the incidence rates of 90-day readmission for those living in neighbourhoods with high migrant density increased for all groups. Swedish-born individuals living in neighbourhoods with high migrant density had 1.29 times (95% CI 1.23 to 1.34), Western migrants had 1.25 times (95% CI 1.12 to 1.40) and non-Western migrants had 1.26 times (IRR=1.26, 95% CI 1.03 to 1.55), a higher rate of readmission compared with those living in neighbourhoods with low migrant density. Figure 1 describes the forest plots of associations.

**Table 4:** Association between neighbourhood migrant density and the 90-day readmission among hospitalised patients with cancer, before the pandemic and during the pandemic

	Before the pandemic (2014–2019)			During the pandemic (2020–2021)		
	Model 1	Model 2	P for interaction	Model 1	Model 2	P for interaction
For Swedish-born						
Low	Ref	Ref	Ref	Ref	Ref	Ref
Medium	1.06 (1.04 to 1.08)*	1.07 (1.05 to 1.09)*	Ref	1.11 (1.08 to 1.15)*	1.14 (1.10 to 1.18)*	Ref
High	1.13 (1.10 to 1.16)*	1.16 (1.13 to 1.19)*	Ref	1.18 (1.14 to 1.22)*	1.29 (1.23 to 1.34)*	Ref
For Western migrants						
Low	Ref	Ref	Ref	Ref	Ref	Ref
Medium	1.07 (1.00 to 1.14)	1.08 (1.01 to 1.16)*	1.00	1.11 (1.01 to 1.23)*	1.13 (1.02 to 1.25)*	0.82
High	1.09 (1.03 to 1.16)*	1.14 (1.07 to 1.22)*	0.89	1.06 (0.97 to 1.17)	1.25 (1.12 to 1.40)*	0.40
For non-Western migrants						
Low	Ref	Ref	Ref	Ref	Ref	Ref
Medium	1.06 (0.90 to 1.25)	1.06 (0.90 to 1.25)	0.23	1.21 (0.99 to 1.48)	1.26 (1.03 to 1.54)*	0.06
High	1.07 (0.92 to 1.24)	1.09 (0.93 to 1.27)	0.73	1.15 (0.96 to 1.39)	1.26 (1.031.55)*	0.72

Model 1: adjusted for age, sex, disposable income, education, civil status and comorbidity index.

Model 2: model 1+area level deprivation index.

Neighbourhood migrant density was calculated by dividing the population of each group in a specific geographic area by the total population of that area.

* P value <0.05.

Online supplemental table 3 describes associations between Western and non-Western migrant density and the 90-day readmission of patients with cancer in each population. Online supplemental tables 4–7 describe age-stratified associations between neighbourhood migrant density and the outcomes among individuals younger than 65 and those aged 65 years and older. Statistically significant associations for mortality were primarily observed among individuals aged 65 years and older.

## Discussion

Using Swedish registers, this study identified that high neighbourhood migrant density increased the rate of 90-day mortality and readmission of Swedish-born and Western migrant patients with cancer, but there was no statistically significant association for non-Western migrants before the pandemic. During the pandemic, the rate for 90-day mortality increased among Western migrants in neighbourhoods with high migrant density. The rates of 90-day readmission increased for all groups in neighbourhoods with high migrant density and the association between neighbourhood migrant density and readmission became significant for non-Western migrants during the pandemic.

A previous study reported that increased mortality of Swedish-born individuals living in migrant-dense neighbourhoods was primarily due to the neighbourhood deprivation.^26^ However, in our study, the higher mortality remained after adjustment for socioeconomic deprivation of neighbourhoods. This discrepancy may be because our study focused on the mortality among hospitalised patients with cancer, who are particularly vulnerable to factors beyond economic deprivation. For patients with cancer, timely access to quality care and adequate follow-up are critical for mortality.^20^ Although Sweden provides universally accessible, high-quality care, regional disparities for the provision of palliative care and specialist therapy were present, particularly in socioeconomically disadvantaged areas.^30 31^ Walk-in centres were established to promote easier access in migrant-dense neighbourhoods, but the disparities were still significant among Swedish-born individuals who prefer traditional healthcare, such as scheduled appointments and access to well-equipped healthcare facilities.^32^ Further research is needed to study the confounding effects of the physical environment on patients with cancer.

Western migrants residing in neighbourhoods with high migrant density experienced higher rates of 90-day mortality and readmission. According to the Swedish Cancer Registry, late-stage cancers were more frequently diagnosed in deprived neighbourhoods, which often overlap with migrant-dense neighbourhoods.^1^ Western migrants had higher incidences of lung, liver and stomach cancers, which are often diagnosed late, and had a higher mortality rate.^4^ When late diagnosis is augmented by barriers to healthcare access in neighbourhoods with high migrant density, the risk of mortality may be further amplified. While our current analysis did not stratify by cancer type, future studies could explore this further.

There were no significant associations between neighbourhood migrant density and mortality and readmission among non-Western migrants before the pandemic. However, we observed a significant interaction indicating that associations of migrant density differed between non-Western migrants and Swedish-born individuals, suggesting that the neighbourhood migrant density operates differently across population groups. Previous studies have also found that ethnic density can be protective against adverse health outcomes, due to strong social bonds and community support networks.^26 33 34^ In Sweden, non-Western migrants have also been reported to experience favourable health outcomes when residing in the high migrant density neighbourhoods.^35 36^ In cancer care, social support may provide tangible resources and better coping strategies, which promote timely seeking care and reduce mortality and readmission following cancer-related hospitalisation.^37^ Nevertheless, as our registry data do not include information on social ties and community support, no definitive conclusions can be reached regarding these.

Relative to the rates before the pandemic, 90-day mortality decreased among Swedish-born individuals but increased among migrants during the pandemic. The reduced mortality among Swedish-born individuals is in accordance with national trends in Sweden, which shows increasing cancer incidence but decreasing mortality, even during the pandemic possibly due to the expanded screening coverage and high-quality care.^38^ Notably, the previously observed interaction between migrant density and migrant status was no longer significant during the pandemic, showing the possible disruption of different associations across migrant groups. Moreover, the readmission rates increased for all groups in neighbourhoods with high migrant density. Higher associations of mortality and readmission within the migrant-dense neighbourhoods during the pandemic can be explained by two pathways. First, since COVID-19 infection and hospitalisation rates were higher in neighbourhoods with high migrant density,^39^ scheduled appointments of patients with cancer were significantly disrupted in these neighbourhoods, thereby limiting their access to adequate treatment and rehabilitation care, leading to an increased need for readmission.^40^ Second, heightened fear of contracting the virus or transmitting it to others while accessing healthcare might have contributed to delayed cancer diagnoses and treatment and higher mortality rates among migrants during the pandemic.^41 42^

Our study has several limitations. First, we assessed the baseline neighbourhood characteristics only for the first year of the study. We could not account for individuals who moved to neighbourhoods with different migrant density or socioeconomic status during the follow-up years due to the complexity and the size of the dataset. Second, since the aim is to compare the overall cancer outcomes of hospitalised patients before and during the pandemic, we did not distinguish between different cancer types, although cancer mortality can vary significantly by type.^3^ Third, we were unable to account for detailed diagnosis and treatment types received by patients, which are the important determinants of the mortality and readmission of hospitalised patients with cancer, as this information was not available in our data. Fourth, our study included only hospitalised patients with cancer, excluding individuals who never reached hospitalisations, particularly non-Western migrants, who tend to have lower healthcare utilisation. Consequently, the study may underestimate the burden of delayed diagnosis among migrants. Finally, since we used the national registry data, we did not include information on the individual health behaviours, body mass index or information on the geographical location of healthcare services, nor how far patients had to travel to access them. These factors can be potential confounders for the associations.

However, this study is the first to examine the associations between neighbourhood migrant density and outcomes of hospitalised patients with cancer. We used nationwide register data covering all regions in Sweden for a comprehensive analysis, which accounts for both individual-level and area-level variations. This study demonstrated clear disparities in cancer outcomes across neighbourhoods with different migrant densities, with increased risks of mortality and readmission among hospitalised patients with cancer in neighbourhoods with high migrant density, both before and during the pandemic. Although Swedish universal healthcare is among the most accessible in the world,^43^ there is a need to improve cancer hospitalisation care for residents in neighbourhoods with high migrant density. Cancer care resources should be allocated to neighbourhoods with high migrant density to invest in community health workers and interpreters, as well as in improving community-based support, to promote after-discharge care and bridge the gap between hospital and home care. During crises, pandemic preparedness plans are necessary to address vulnerable populations in marginalised neighbourhoods. These plans should include developing cancer care pathways and community outreach programmes to address the fear of the pandemic, and they could be adapted globally. Moreover, further research is required to explore how high infection rates during the pandemic influenced the health-seeking behaviours of individuals with chronic diseases in these neighbourhoods.

## Conclusion

Swedish-born individuals and migrants in neighbourhoods with high migrant density experienced higher mortality and readmissions after cancer hospitalisation. Health disparities between neighbourhoods of different migrant density became more evident during the pandemic. Further studies are needed to determine whether these findings apply to different types of cancer. Access to cancer-related healthcare needs to be improved in migrant dense neighbourhoods, both as an ongoing effort and as a preparation for future pandemics.

## Supplementary material

10.1136/bmjopen-2025-113681 online supplemental file 1

## Data Availability

Data may be obtained from a third party and are not publicly available.

## References

[1] Cancer in sweden-statistics & facts. https://www.statista.com/topics/9392/cancer-in-sweden/#topicOverview.

[2] Dhaher NF, Pikkemaat M, Shaat N (2023). Cancer, cardiovascular disease, and all-cause mortality in Iraqi- and Swedish-born individuals in Sweden: the MEDIM cohort study. Sci Rep.

[3] Tollosa DN, Zendehdel K, Procopio A (2024). Cancer mortality by country of birth and cancer type in Sweden: A 25-year registry-based cohort study. Cancer Med.

[4] Tollosa DN, Zendehdel K, Boffetta P (2025). Disparities in overall and site-specific cancer mortality among immigrant generations in Sweden: a nationwide follow-up study over 3 decades. Am J Epidemiol.

[5] Rostila M, Fritzell J (2014). Mortality differentials by immigrant groups in Sweden: the contribution of socioeconomic position. Am J Public Health.

[6] Ullgren H, Sharp L, Olofsson A (2021). Factors associated with healthcare utilisation during first year after cancer diagnose-a population-based study. Eur J Cancer Care (Engl).

[7] Willén L, Berglund A, Bergström S (2022). Patterns of care and outcomes in immigrants with non-small cell lung cancer. A population-based study (Sweden). PLoS One.

[8] Mousavi SM, Hemminki K (2015). Cancer incidence, trends, and survival among immigrants to Sweden: a population-based study. Eur J Cancer Prev.

[9] New statistics on integration and migration in the Nordics. https://www.nordicstatistics.org/news/new-statistics-on-integration-and-migration-in-the-nordics/.

[10] Dunlavy A (2017). Between two worlds: studies of migration, work, and health.

[11] Andersson R, Kährik A (2015). Widening gaps: segregation dynamics during two decades of economic and institutional change in stockholm. socio-economic segregation in european capital cities.

[12] Esaiasson P, Sohlberg J (2025). Social order in Sweden’s politicized and vulnerable neighborhoods – The perspective of residents. Eur J Polit Res.

[13] Gustafsson B, Österberg T (2023). In and out of privileged and disadvantaged neighbourhoods in Sweden: On the importance of country of birth. Population Space and Place.

[14] Wilkens J, Thulesius H, Schmidt I (2016). The 2015 National Cancer Program in Sweden: Introducing standardized care pathways in a decentralized system. Health Policy.

[15] Burström B, Burström K, Nilsson G (2017). Equity aspects of the Primary Health Care Choice Reform in Sweden - a scoping review. Int J Equity Health.

[16] Kawakami N, Winkleby M, Skog L (2011). Differences in neighborhood accessibility to health-related resources: A nationwide comparison between deprived and affluent neighborhoods in Sweden. Health Place.

[17] Wixe S, Lobo J, Mellander C (2024). Evidence of COVID-19 fatalities in Swedish neighborhoods from a full population study. Sci Rep.

[18] Nwaru C, Bonander C, Li H (2025). Neighbourhood immigrant density and COVID-19 infection and hospitalisation among healthcare workers in Sweden: a register-based observational study. BMJ Public Health.

[19] Chakravarty D, Ratnani P, Sobotka S (2021). Increased Hospitalization and Mortality from COVID-19 in Prostate Cancer Patients. Cancers (Basel).

[20] Li X, Sundquist J, Zöller B (2015). Neighborhood deprivation and lung cancer incidence and mortality: a multilevel analysis from Sweden. J Thorac Oncol.

[21] Ludvigsson JF, Almqvist C, Bonamy A-KE (2016). Registers of the Swedish total population and their use in medical research. Eur J Epidemiol.

[22] Ludvigsson JF, Svedberg P, Olén O (2019). The longitudinal integrated database for health insurance and labour market studies (LISA) and its use in medical research. Eur J Epidemiol.

[23] Ludvigsson JF, Andersson E, Ekbom A (2011). External review and validation of the Swedish national inpatient register. BMC Public Health.

[24] Miething A, Juárez SP (2024). Income mortality paradox by immigrants’ duration of residence in Sweden: a population register-based study. J Epidemiol Community Health.

[25] Miething A, Dunlavy A, Juárez SP (2025). Income inequalities and mortality by generation among individuals with a foreign background in Sweden: a population-based study. *Lancet Reg Health Eur*.

[26] Cederström A, Dunlavy A (2025). A matter of measurement? A Swedish register-based study of migrant residential segregation and all-cause mortality. SSM Popul Health.

[27] DeSO in the statistics database. https://www.scb.se/hitta-statistik/regional-statistik-och-kartor/regionala-indelningar/deso---demografiska-statistikomraden/deso-tabellerna-i-ssd--information-och-instruktioner/.

[28] NCI comorbidity index overview. https://healthcaredelivery.cancer.gov/seermedicare/considerations/comorbidity.html#:~:text=The%20NCI%20Comorbidity%20Index%20excludes,further%20consolidation%20to%2014%20conditions.

[29] Musca SC, Kamiejski R, Nugier A (2011). Data with hierarchical structure: impact of intraclass correlation and sample size on type-I error. Front Psychol.

[30] Ozanne A, Öhlén J, Nyblom S (2025). Disparities in end-of-life care and place of death in people with malignant brain tumors-A Swedish registry study. Neurooncol Pract.

[31] (2025). EU country cancer profile: Sweden. https://www.oecd.org/en/publications/eu-country-cancer-profile-sweden-2025_39c18d93-en.html.

[32] Wärdig R, Hadziabdic E, Hjelm K (2021). Evaluation of a healthcare walk-in centre in an immigrant-dense area from the perspective of Swedish-born patients. Prim Health Care Res Dev.

[33] Bécares L, Shaw R, Nazroo J (2012). Ethnic density effects on physical morbidity, mortality, and health behaviors: a systematic review of the literature. Am J Public Health.

[34] Schofield H, Hill RM, Feys O (2024). A novel, robust, and portable platform for magnetoencephalography using optically-pumped magnetometers. Imaging Neurosci (Camb).

[35] Dykxhoorn J, Lewis G, Hollander A-C (2020). Association of neighbourhood migrant density and risk of non-affective psychosis: a national, longitudinal cohort study. Lancet Psychiatry.

[36] Mezuk B, Cederin K, Li X (2014). Immigrant enclaves and risk of diabetes: a prospective study. BMC Public Health.

[37] Ochoa-Dominguez CY, Chan RY, Cervantes L (2024). Social support experiences of hispanic/latino parents of childhood cancer survivors in a safety-net hospital: a qualitative study. J Psychosoc Oncol.

[38] (2023). Statistics on cancer incidence.

[39] Rostila M, Cederström A, Wallace M (2023). Inequalities in COVID-19 severe morbidity and mortality by country of birth in Sweden. Nat Commun.

[40] Jones D, Neal RD, Duffy SRG (2020). Impact of the COVID-19 pandemic on the symptomatic diagnosis of cancer: the view from primary care. Lancet Oncol.

[41] Luo Q, O’Connell DL, Yu XQ (2022). Cancer incidence and mortality in Australia from 2020 to 2044 and an exploratory analysis of the potential effect of treatment delays during the COVID-19 pandemic: a statistical modelling study. Lancet Public Health.

[42] Lofters AK, Wu F, Frymire E (2023). Cancer Screening Disparities Before and After the COVID-19 Pandemic. JAMA Netw Open.

[43] Wagstaff A, Neelsen S (2020). A comprehensive assessment of universal health coverage in 111 countries: a retrospective observational study. Lancet Glob Health.

