# Supplementary material for: Neighbourhood migrant density and outcomes in hospitalised patients with cancer before and during the COVID-19 pandemic in Sweden: a register-based retrospective cohort study
Source: BMJ Open. 2026 May 18;16(5):e113681. doi: 10.1136/bmjopen-2025-113681 (PMC13185031; doi:10.1136/bmjopen-2025-113681)
Supplement: online supplemental file 1 [file bmjopen-16-5-s001.docx]

| **Supplementary Table 1: Neighbourhood migrant density and deprivation index of the DeSOs residing in the first years of study** | | | | | | | | |
| --- | --- | --- | --- | --- | --- | --- | --- | --- |
|  | **Before the pandemic (2014)** | | | | **During the pandemic (2020)** | | | |
|  | **Total** | **Swedish-born** | **Western migrants** | **Non-Western migrants** | **Total** | **Swedish-born** | **Western migrants** | **Non-Western migrants** |
| **Migrant density** |  |  |  |  |  |  |  |  |
| Low | 87,890 (36.1%) | 82,403 (39.2%) | 4,759 (19.8%) | 728 (8.0%) | 42,147 (37.3%) | 39,436 (41.1%) | 2,260 (20.1%) | 451 (8.0%) |
| Medium | 83,890 (34.5%) | 74,445 (35.4%) | 7,608 (31.7%) | 1,837 (20.1%) | 38,657 (34.2%) | 33,828 (35.2%) | 3,637 (32.3%) | 1,192 (21.2%) |
| High | 71,577 (29.4%) | 53,385 (25.4%) | 11,617 (48.4%) | 6,575 (71.9%) | 32,131 (28.5%) | 22,787 (23.7%) | 5,374 (47.7%) | 3,970 (70.7%) |
| **Western migrant density** |  |  |  |  |  |  |  |  |
| Low | 86,392 (35.5%) | 81,027 (38.5%) | 4,246 (17.7%) | 1,119 (12.2%) | 40,640 (36.0%) | 37,763 (39.3%) | 2,071 (18.4%) | 806 (14.4%) |
| Medium | 83,297 (34.2%) | 73,320 (34.8%) | 7,525 (31.4%) | 2,452 (26.8%) | 38,419 (34.0%) | 33,382 (34.8%) | 3,492 (31.0%) | 1,545 (27.5%) |
| High | 73,668 (30.3%) | 55,886 (26.6%) | 12,213 (50.9%) | 5,569 (60.9%) | 33,876 (30.0%) | 24,906 (25.9%) | 5,708 (50.6%) | 3,262 (58.1%) |
| **Non-Western migrant density** |  |  |  |  |  |  |  |  |
| Low | 87,936 (36.1%) | 81,593 (38.8%) | 5,639 (23.5%) | 704 (7.7%) | 42,477 (37.6%) | 39,365 (41.0%) | 2,708 (24.0%) | 404 (7.2%) |
| Medium | 83,206 (34.2%) | 73,726 (35.1%) | 7,649 (31.9%) | 1,831 (20.0%) | 38,169 (33.8%) | 33,445 (34.8%) | 3,509 (31.1%) | 1,215 (21.7%) |
| High | 72,215 (29.7%) | 54,914 (26.1%) | 10,696 (44.6%) | 6,605 (72.3%) | 32,289 (28.6%) | 23,241 (24.2%) | 5,054 (44.8%) | 3,994 (71.2%) |
| **Socioeconomic Deprivation Index (Decile)** |  |  |  |  |  |  |  |  |
| D1 | 20,956 (8.6%) | 18,610 (8.9%) | 1,835 (7.7%) | 511 (5.6%) | 10,732 (9.5%) | 9,569 (10.0%) | 863 (7.7%) | 300 (5.3%) |
| D2 | 23,361 (9.6%) | 20,972 (10.0%) | 1,927 (8.0%) | 462 (5.1%) | 11,208 (9.9%) | 9,992 (10.4%) | 923 (8.2%) | 293 (5.2%) |
| D3 | 24,624 (10.1%) | 21,990 (10.5%) | 2,119 (8.8%) | 515 (5.6%) | 12,002 (10.6%) | 10,733 (11.2%) | 948 (8.4%) | 321 (5.7%) |
| D4 | 25,759 (10.6%) | 22,941 (10.9%) | 2,174 (9.1%) | 644 (7.1%) | 11,896 (10.5%) | 10,694 (11.1%) | 900 (8.0%) | 302 (5.4%) |
| D5 | 26,282 (10.8%) | 23,284 (11.1%) | 2,265 (9.4%) | 733 (8.0%) | 12,570 (11.1%) | 11,221 (11.7%) | 1,023 (9.1%) | 326 (5.8%) |
| D6 | 27,284 (11.2%) | 23,86 (11.4%) | 2,544 (10.6%) | 880 (9.6%) | 12,590 (11.2%) | 11,042 (11.5%) | 1,160 (10.3%) | 388 (6.9%) |
| D7 | 26,751 (11.0%) | 22,961 (10.9%) | 2,686 (11.2%) | 1,104 (12.1%) | 12,781 (11.3%) | 11,047 (11.5%) | 1,193 (10.6%) | 541 (9.6%) |
| D8 | 27,084 (11.1%) | 23,045 (11.0%) | 2,880 (12.0%) | 1,159 (12.7%) | 12,284 (10.9%) | 10,094 (10.5%) | 1,429 (12.7%) | 761 (13.6%) |
| D9 | 27,312 (11.2%) | 22,299 (10.6%) | 3,376 (14.1%) | 1,637 (17.9%) | 11,767 (10.4%) | 8,901 (9.3%) | 1,737 (15.4%) | 1,129 (20.1%) |
| D10 | 13,944 (5.7%) | 10,271 (4.9%) | 2,178 (9.1%) | 1,495 (16.4%) | 5,105 (4.5%) | 2,758 (2.9%) | 1,095 (9.7%) | 1,252 (22.3%) |

DeSO: Demografiska statistikområden

Neighbourhood migrant density was calculated by dividing the population of each group in a specific geographic area by the total population of that area.

| **Supplementary Table 2: Association between neighbourhood Western and non-Western migrant density and the 90-day mortality among hospitalized patients with cancer, before the pandemic and during the pandemic** | | | | | |
| --- | --- | --- | --- | --- | --- |
|  | Before the pandemic (2014-2019) | | During the pandemic (2020-2021) | | |
|  | Model 1 | Model 2 | Model 1 | Model 2 | |
| **For Swedish-born** |  |  |  |  | |
| Western Density |  |  |  |  | |
| Low | ref | ref | ref | ref | |
| Medium | **1.04 (1.01, 1.07)** | **1.04 (1.01, 1.07)** | 1.03 (0.99, 1.07) | 1.02 (0.98, 1.06) | |
| High | **1.14 (1.11, 1.17)** | **1.14 (1.10, 1.17)** | **1.08 (1.04, 1.13)** | **1.06 (1.02, 1.11)** | |
| Non-Western density |  |  |  |  | |
| Low | ref | ref | ref | ref | |
| Medium | **1.05 (1.02, 1.08)** | **1.05 (1.02, 1.07)** | 1.05 (1.01, 1.09) | **1.05 (1.00, 1.09)** | |
| High | **1.12 (1.09, 1.15)** | **1.12 (1.08, 1.15)** | **1.10 (1.05, 1.15)** | **1.07 (1.02, 1.13)** | |
| **For Western migrants** |  |  |  |  | |
| Western Density |  |  |  |  | |
| Low | ref | ref | ref | ref | |
| Medium | 1.03 (0.94, 1.12) | 1.03 (0.94, 1.12) | 1.03 (0.90, 1.17) | 1.03 (0.91, 1.18) | |
| High | **1.10 (1.02, 1.19)** | **1.11 (1.02, 1.20)** | **1.14 (1.01, 1.29)** | **1.17 (1.03, 1.32)** | |
| Non-Western density |  |  |  |  | |
| Low | ref | ref | ref | ref | |
| Medium | 0.95 (0.88, 1.03) | 0.95 (0.88, 1.03) | 1.10 (0.97, 1.23) | **1.11 (0.99, 1.26)** | |
| High | 1.03(0.96, 1.10) | 1.03 (0.95, 1.11) | 1.10 (0.98, 1.23) | **1.17 (1.02, 1.33)** | |
| **For Non-Western migrants** |  |  |  |  |  |
| Western Density |  |  |  |  | |
| Low | ref | ref | ref | ref | |
| Medium | 1.03 (0.85, 1.24) | 1.02 (0.85, 1.23) | 0.98 (0.76, 1.26) | 0.99 (0.77, 1.26) | |
| High | 0.95 (0.80, 1.12) | 0.92 (0.77, 1.09) | 1.04 (0.82, 1.30) | 0.99 (0.79, 1.25) | |
| Non-Western density |  |  |  |  | |
| Low | ref | ref | ref | ref | |
| Medium | 0.81 (0.64,1.03) | 0.79 (0.63, 1.01) | 1.17 (0.82, 1.67) | 1.17 (0.82, 1.68) | |
| High | 0.93 (0.75, 1.14) | 0.89 (0.71, 1.11) | 1.17 (0.84, 1.63) | 0.96 (0.67, 1.40) | |
| Model 1: Adjusted for age, sex, disposable income, education, civil status and comorbidity index. | | | | | |
| Model 2: Model 1+ Area level deprivation index.  Neighbourhood migrant density was calculated by dividing the population of each group in a specific geographic area by the total population of that area. | | | | | |

| **Supplementary Table 3: Association between neighbourhood Western and non-Western migrant density and the 90-day readmission among hospitalized patients with cancer, before the pandemic and during the pandemic** | | | | | | | | | | |
| --- | --- | --- | --- | --- | --- | --- | --- | --- | --- | --- |
|  | Before the pandemic (2014-2019) | | | | | During the pandemic (2020-2021) | | | | |
|  | Model 1 | | Model 2 | | | Model 1 | | | Model 2 | |
| **For Swedish-born** |  | |  | | |  | | |  | |
| Western Density |  | |  | | |  | | |  | |
| Low | ref | | ref | | | ref | | | ref | |
| Medium | **1.03 (1.01, 1.06)** | | **1.04 (1.01, 1.06)** | | | **1.07 (1.03, 1.10)** | | | **1.07 (1.03, 1.11)** | |
| High | **1.14 (1.11, 1.17)** | | **1.14 (1.12, 1.17)** | | | **1.19 (1.15, 1.24)** | | | **1.22 (1.17, 1.26)** | |
| Non-Western density |  | | | |  | | |  | |  |
| Low | ref | | ref | | | ref | | | ref | |
| Medium | **1.04 (1.01, 1.06)** | | **1.05 (1.03, 1.08)** | | | **1.05 (1.02, 1.09)** | | | **1.09 (1.04, 1.13)** | |
| High | **1.08 (1.06, 1.11)** | | **1.12 (1.09, 1.15)** | | | **1.14(1.10, 1.18)** | | | **1.26 (1.20, 1.32)** | |
| **For Western migrants** |  | |  | | |  | | |  | |
| Western Density |  | |  | | |  | | |  | |
| Low | ref | | ref | | | ref | | | ref | |
| Medium | 1.04 (0.97, 1.12) | | 1.04 (0.97, 1.12) | | | **1.13 (1.02, 1.25)** | | | **1.13 (1.01, 1.25)** | |
| High | **1.15 (1.08, 1.23)** | | **1.18 (1.10, 1.26)** | | | **1.15 (1.05, 1.27)** | | | **1.24 (1.12, 1.37)** | |
| Non-Western density | | |  | | |  | | |  | |
| Low | ref | | ref | | | ref | | | ref | |
| Medium | 1.00 (0.94, 1.07) | | 1.02 (0.95, 1.09) | | | 1.06 (0.97, 1.17) | | | 1.10 (1.00, 1.22) | |
| High | 1.01 (0.95, 1.08) | | 1.05 (0.98, 1.12) | | | 1.00 (0.92, 1.10) | | | 1.18 (1.06, 1.32) | |
| **For Non-Western migrants** |  |  | |  | | |  | | | |
| Western Density |  | |  | | |  | | |  | |
| Low | ref | | ref | | | ref | | | ref | |
| Medium | 1.06 (0.93, 1.22) | | 1.06 (0.93, 1.21) | | | 1.16 (0.99, 1.36) | | | 1.16 (0.99, 1.35) | |
| High | 1.02 (0.90, 1.15) | | 1.01 (0.90, 1.15) | | | 1.13 (0.97, 1.30) | | | 1.15 (0.99, 1.33) | |
| Non-Western density | | |  | | |  | | |  | |
| Low | ref | | ref | | | ref | | | ref | |
| Medium | 0,96 (0.82, 1.13) | | 0.95 (0.81, 1.12) | | | 0.96 (0.79, 1.16) | | | 1.01 (0.83, 1.22) | |
| High | 1.05 (0.91, 1.21) | | 1.08 (0.92, 1.26) | | | 1.00 (0.83, 1.19) | | | 1.13 (0.93, 1.39) | |
| Model 1: Adjusted for age, sex, disposable income, education, civil status and comorbidity index. | | | | | | | | | | |
| Model 2: Model 1+ Area level deprivation index  Neighbourhood migrant density was calculated by dividing the population of each group in a specific geographic area by the total population of that area. | | | | | | | | | | |

| **Supplementary Table 4: Association between neighborhood migrant density and the 90-days mortality for cancer hospitalization (Below 65 years old)** | | | | |
| --- | --- | --- | --- | --- |
|  | Before COVID (2014-2019) (N=81,552) | | During COVID (2020-2021) (N=37,095) | |
|  | Model 1 | Model 2 | Model 1 | Model 2 |
| **For Swedish** |  |  |  |  |
| Migrant density |  |  |  |  |
| Low | ref | ref | ref | ref |
| Medium | 0. 97 (0.92, 1.02) | 0.95 (0.91, 1.00) | 0.93 (0.85, 1.02) | 0.90 (0.82, 0.98) |
| High | 1.04 (0.98, 1.09) | 1.00 (0.95, 1.06) | 1.03 (0.93, 1.13) | 0.89 (0.80, 1.00) |
| **For Western migrants** |  |  |  |  |
| Migrant density |  |  |  |  |
| Low | ref | ref | ref | ref |
| Medium | 0.99 (0.84, 1.16) | 0.96 (0.82, 1.14) | 1.16 (0.87, 1.53) | 1.11 (0.83, 1.47) |
| High | 1.00 (0.86, 1.15) | 0.94 (0.80, 1.10) | 1.26 (0.97, 1.63) | 1.23 (0.92, 1.67) |
| **For Non-Western migrants** |  |  |  |  |
| Migrant density |  |  |  |  |
| Low | ref | ref | ref | ref |
| Medium | 0.91 (0.69, 1.21) | 0.89 (0.67, 1.18) | 0.78 (0.52, 1.18) | 0.81 (0.53, 1.22) |
| High | 0.90 (0.71, 1.15) | 0.86 (0.66, 1.11) | 0.93 (0.65, 1.33) | 0.74 (0.50, 1.11) |
| Model 1: Adjusted for age, sex, disposable income, education, marital status and comorbidity index. | | | | |
| Model 2: Model 1+ Area level deprivation index | | | | |

| **Supplementary Table 5: Association between neighborhood migrant density and the 90-days mortality for cancer hospitalization (Above 65 years old)** | | | | |
| --- | --- | --- | --- | --- |
|  | Before COVID (2014-2019)  (N=161, 805) | | During COVID (2020-2021) (N=75,840) | |
|  | Model 1 | Model 2 | Model 1 | Model 2 |
| **For Swedish** |  |  |  |  |
| Migrant density |  |  |  |  |
| Low | ref | ref | ref | ref |
| Medium | **1.07 (1.03, 1.10)** | **1.07 (1.04, 1.10)** | **1.06 (1.01, 1.10)** | **1.06 (1.02, 1.11)** |
| High | **1.19 (1.15, 1.22)** | **1.20 (1.16, 1.24)** | **1.10 (1.05, 1.15)** | **1.10 (1.04, 1.16)** |
| **For Western migrants** |  |  |  |  |
| Migrant density |  |  |  |  |
| Low | ref | ref | ref | ref |
| Medium | 0.98 (0.89, 1.08) | 0.99 (0.90, 1.09) | 1.09 (0.95, 1.26) | 1.13 (0.97, 1.30) |
| High | **1.10 (1.01, 1.20)** | **1.14 (1.04, 1.25)** | **1.13 (0.99, 1.29)** | **1.24 (1.07, 1.45)** |
| **For Non-Western migrants** |  |  |  |  |
| Migrant density |  |  |  |  |
| Low | ref | ref | ref | ref |
| Medium | 0.86 (0.57, 1.31) | 0.86 (0.56, 1.31) | 1.78 (0.97, 3.27) | **1.81 (1.00, 3.28)** |
| High | 0.98 (0.68, 1.42) | 0.95 (0.63, 1.44) | 1.50 (0.84, 1.68) | 1.28 (0.68, 2.39) |
| Model 1: Adjusted for age, sex, disposable income, education, marital status and comorbidity index. | | | | |
| Model 2: Model 1+ Area level deprivation index | | | | |

| **Supplementary Table 6: Association between neighborhood migrant density and the 90-days readmission for cancer hospitalization (Below 65 years old)** | | | | |
| --- | --- | --- | --- | --- |
|  | Before COVID (2014-2019) (N=81,552) | | During COVID (2020-2021)  (N=37,095) | |
|  | Model 1 | Model 2 | Model 1 | Model 2 |
| **For Swedish** |  |  |  |  |
| Migrant density |  |  |  |  |
| Low | ref | ref | ref | ref |
| Medium | 1.00 (0.97, 1.04) | 0.99 (0.96, 1.03) | **1.12 (1.06, 1.18)** | **1.13 (1.07, 1.19)** |
| High | 1.02 (0.98, 1.06) | 1.00 (0.96, 1.04) | **1.13 (1.06, 1.20)** | **1.21 (1.13, 1.30)** |
| **For Western migrants** |  |  |  |  |
| Migrant density |  |  |  |  |
| Low | ref | ref | ref | ref |
| Medium | 1.04 (0.92, 1.17) | 1.04 (0.92, 1.17) | **1.27 (1.06, 1.52)** | **1.29 (1.07, 1.55)** |
| High | 1.02 (0.91, 1.14) | 1.04 (0.92, 1.17) | 1.10 (0.93, 1.31) | **1.31 (1.07, 1.60)** |
| **For Non-Western migrants** |  |  |  |  |
| Migrant density |  |  |  |  |
| Low | ref | ref | ref | ref |
| Medium | 0.98 (0.81, 1.18) | 0.98 (0.81, 1.18) | **1.27 (1.00, 1.63)** | **1.31 (1.02, 1.68)** |
| High | 0.99 (0.84, 1.17) | 1.01 (0.85, 1.20) | 1.24 (0.99, 1.56) | **1.36 (1.06, 1.74)** |
| Model 1: Adjusted for age, sex, disposable income, education, marital status and comorbidity index. | | | | |
| Model 2: Model 1+ Area level deprivation index | | | | |

| **Supplementary Table 7: Association between neighborhood migrant density and the 90-days readmission for cancer hospitalization (Above 65 years old)** | | | | |
| --- | --- | --- | --- | --- |
|  | Before COVID (2014-2019)  (N=161, 805) | | During COVID (2020-2021)  (N=75,840) | |
|  | Model 1 | Model 2 | Model 1 | Model 2 |
| **For Swedish** |  |  |  |  |
| Migrant density |  |  |  |  |
| Low | ref | ref | ref | ref |
| Medium | **1.09 (1.06, 1.12)** | **1.11 (1.08, 1.14)** | **1.11 (1.06, 1.15)** | **1.14 (1.09, 1.19)** |
| High | **1.18 (1.15, 1.22)** | **1.24 (1.20, 1.28)** | **1.19 (1.14, 1.24)** | **1.31 (1.24, 1.38)** |
| **For Western migrants** |  |  |  |  |
| Migrant density |  |  |  |  |
| Low | ref | ref | ref | ref |
| Medium | **1.08 (1.00, 1.17)** | **1.10 (1.01, 1.19)** | 1.05 (0.93, 1.18) | 1.07 (0.94, 1.20) |
| High | **1.13 (1.05, 1.22)** | **1.19 (1.10, 1.29)** | 1.05 (0.93, 1.17) | **1.23 (1.08, 1.40)** |
| **For Non-Western migrants** |  |  |  |  |
| Migrant density |  |  |  |  |
| Low | ref | ref | ref | ref |
| Medium | 1.43 (0.97, 2.10) | 1.39 (0.95, 2.05) | 1.06 (0.75, 1.52) | 1.10 (0.77, 1.57) |
| High | 1.41 (0.98, 2.02) | 1.44 (0.98, 2.11) | 0.94 (0.67, 1.30) | 1.02 (0.71, 1.47) |
| Model 1: Adjusted for age, sex, disposable income, education, marital status and comorbidity index. | | | | |
| Model 2: Model 1+ Area level deprivation index | | | | |
