# Supplementary material for: Neighbourhood migrant density and outcomes in hospitalised patients with cancer before and during the COVID-19 pandemic in Sweden: a register-based retrospective cohort study
Source: BMJ Open. 2026 May 18;16(5):e113681. doi: 10.1136/bmjopen-2025-113681 (PMC13185031; doi:10.1136/bmjopen-2025-113681)
Supplement: Supplementary file 2 [file bmjopen-2025-113681.reviewer_comments.pdf]

PEER REVIEW HISTORY

BMJ Open publishes all reviews undertaken for accepted manuscripts. Reviewers are asked to complete a checklist review form and are provided with free text boxes to elaborate on their assessment. These free text comments are reproduced below.

ARTICLE DETAILS

Title (Provisional)

Neighbourhood migrant density and outcomes in hospitalized patients with cancer before and during the COVID-19 pandemic in Sweden: A register-based retrospective cohort study

Authors

Khin, Yu Par; Fujiwara, Takeo; Nigusse Tollosa, Daniel; Rostila, Mikael; Miething, Alexander

---

VERSION 1 - REVIEW

**Reviewer**	**1**
**Name**	**Kelson, Zoe**
**Affiliation**	**University of Exeter, Mathematics**
**Date**	**09-Dec-2025**
**COI**	**None**

This retrospective cohort study examined whether neighbourhood migrant density influenced hospitalized cancer patients' outcomes, such as mortality and readmission rates, and whether these associations varied before and during the COVID-19 pandemic.

Reviewer comments:

"Hospitalized cancer patients (ICD code C00-C97) from 2014-2019 (before the pandemic) and 2020-2021 (during the pandemic)"

and

"used multiple longitudinal national registers linked with unique pseudonymized personal identifiers, covering the total population of Sweden. The linked datasets include the total population register, the Longitudinal Integration Database for Health Insurance and Labour Market Studies (LISA), and the National Outpatient and Inpatient Register. "

Can the authors please further comment on the completeness and coverage of this linked data?

"Individuals who had been hospitalized for cancer before the pandemic and survived were eligible to be included in the study during the pandemic period."

Can the authors please conduct a sensitivity analysis on this, only comparing first hospitalisation outcomes for all individuals for example?

"conducted a completecase analysis, excluding individuals with missing data on outcomes, exposures, or covariates as the missing percentage is low (less than 3 percent)."

Can the authors please assess whether missing data can be assumed to be missing at random?

"Individual-level covariates included age (categorized into 5-year intervals), sex (male or female), education level (primary, secondary, or post-secondary), civil status (with or without a partner), the comorbidity index measured in the first year of observation, and the mean disposable income during the study period...

Area-level deprivation for each DeSO unit was included as a time-fixed contextual covariate, measured at the first year of observation"

Can the authors please include BMI in the study?

Is data on lifestyle factors, such as smoking, alcohol, physical activity, available for inclusion?

"multilevel Poisson regression with robust variance, which is appropriate for common outcomes (more than 10 percent). A multilevel model with a random intercept was specified, with individuals nested within DeSO units. The logarithm of follow-up person-years was included as an offset. Analyses were stratified into three population groups: Swedish-born, Western migrants, and non-Western migrants. Incidence Rate Ratios (IRRs) were calculated to estimate the 90- day mortality and 90-day readmission. Model 1 was adjusted for age, sex, disposable income, education level, civil status, and comorbidity index while Model 2 was additionally adjusted for the area-level deprivation index."

Appropriate modelling methods have been applied by the authors.

The Results are reported accurately and the Tables are clear and informative.

"Lack of distinction between different cancer types and stages. • Inclusion of only hospitalized cancer patients excluding those who had delayed diagnosis or never reached hospitalization." [Strengths and Limitations of this Study]

and

"Our study has several limitations. First, we assessed the baseline neighbourhood characteristics only for the first year of the study. We could not account for individuals who moved to neighbourhoods with different migrant density or socioeconomic status during the follow-up years due to the complexity and the size of the dataset. Second, we conducted a complete case analysis and might introduce bias from the missing data. Third, since the aim is to compare the overall cancer outcomes of hospitalized patients before and during

pandemic, we did not distinguish between different cancer types, although cancer mortality can vary significantly by type 3 . Fourth, we were unable to account for the stages of the cancer and types of treatment received by patients, which are the important determinants of the mortality and readmission of hospitalized cancer patients, as this information was not available in our data. Finally, our study included only hospitalized cancer patients, excluding individuals who never reached hospitalizations, particularly non-Western migrants, who tend to have lower healthcare utilization. Consequently, the study may underestimate the burden of delayed diagnosis among migrants. "

A discussion on the study limitations is provided by the authors.

Thanks for providing a copy of the STROBE checklist.

Reviewer **2**

Name **Hajebrahimi , Sakineh**

Affiliation **Iranian Evidence Based Medicine Centre of Excellence, Tabriz University of Medical Sciences, Tabriz, Iran.**

Date **02-Feb-2026**

COI **None**

The manuscript should more explicitly frame "high migrant density" not just as a demographic measure but as a marker of systemic marginalization. It likely correlates with under-resourced primary care, linguistic barriers, lower health literacy, and poorer social determinants of health for all residents. This framing is crucial for interpreting why Swedish-born individuals in these areas are also adversely affected.

The discussion on mechanisms is good but can be sharper. For the pandemic period, consider structuring the discussion around a clear conceptual model:

Direct Pathway: Higher COVID-19 incidence in these neighborhoods → disruption of cancer care, nosocomial infection risk for immunocompromised patients.

Indirect Pathway: Fear of infection, misinformation, strained healthcare capacity → delayed presentations and follow-up → sicker patients at admission and discharge.

The discussion on the potential protective "ethnic density effect" is appropriate. However, to strengthen this, consider formally testing an interaction between individual migrant status (non-Western) and neighborhood non-Western migrant density. Does the (null/moderate) effect of density differ for non-Western migrants versus Swedish-born individuals? This could provide statistical support for the hypothesized protective buffering. Acknowledge that the younger age of non-Western migrants is a major competing explanation for their lower mortality. Could you run a sensitivity analysis age-standardizing the rates or presenting an age-stratified analysis (<65 vs. 65+)?

The analysis presents results for three exposure variables: total, Western, and non-Western migrant density. Which is considered the primary exposure? The rationale for analyzing all three should be stated upfront in the methods. It might be clearer to present total migrant density as the main result in the abstract and core tables, with Western/non-Western breakdowns as stratified or sensitivity analyses.

Consider reporting measures of model fit (e.g., Variance Partition Coefficient - VPC) from your multilevel models. How much of the total variance in outcomes is explained at the neighborhood (DeSO) level? Does adding migrant density and area deprivation substantially reduce this between-area variance?

Forest plots showing IRRs and 95% CIs for "High vs. Low" migrant density for mortality and readmission, stratified by population group (Swedish/Western/Non-Western) and period (Pre/During Pandemic). This would allow readers to instantly grasp the magnitude, precision, and patterns of the main effects.

The discussion should more forcefully propose testable mechanisms for the observed disparities. For example, are there data on the density of oncology clinics, palliative care services, or primary care physicians with cancer expertise across DeSO areas? Even citing related literature would help.

The conclusion is good but can be more specific and actionable. What does "improve cancer care" mean in practice?

Recommendation 1: Targeted resource allocation for hospital discharge planning and community-based support in high-density neighborhoods.

Recommendation 2: Developing pandemic preparedness plans that explicitly address the vulnerability of cancer patients in marginalized neighborhoods (e.g., protected cancer care pathways, community outreach to address fear).

Recommendation 3: Investment in community health workers and interpreters embedded within these neighborhoods to bridge hospital and home care.

Briefly contextualize the findings beyond Sweden. While Sweden's universal system is unique, the clustering of marginalized populations in under-resourced urban areas is a global phenomenon. The lessons about how such area-level vulnerability interacts with a health system shock are universally important.

Minor Revisions:

Abstract Results: The abstract states mortality rates "slightly increased among Western migrants" during the pandemic. The result (IRR: 1.22, 95% CI: 1.07, 1.39) suggests a 22% increase, which is substantial. Consider rephrasing to "significantly increased."

Missing Data: The statement "missing percentage is low (less than 3 percent)" is reassuring. Specify if this is for all variables combined or for key variables. A supplemental table showing patterns of missingness would be ideal.

**Reviewer**	**3**
**Name**	**Sato, Takuya**
**Affiliation**	**The University of Tokyo Hospital, Emergency & Critical Care Medicine**
**Date**	**02-Mar-2026**
**COI**	**None**

Dear Authors,

Thank you for the opportunity to review your manuscript “Neighbourhood migrant density and outcomes in hospitalized cancer patients before and during the COVID-19 pandemic in Sweden: A register-based study”. This retrospective observational cohort study investigated the association between neighborhood migrant density and the mortality after cancer hospitalization. The results demonstrated a significantly higher mortality rate among the Swedish-born population and western immigrants living in high migrant density area. The perspective is novel and interesting. However, I have a few comments and questions that may help clarify the findings for the readers.

1.

The authors state, *"This discrepancy may be because our study focused on the mortality among hospitalized cancer patients, who are particularly vulnerable for factors beyond economic deprivation."* (Page 9, Line 33)

While I agree that cancer patients are highly vulnerable, this statement only explains an individual-level susceptibility, not the neighborhood-level mechanism. It does not explain *why* living in a high-migrant-density area exacerbates this vulnerability even after adjusting for individual and neighborhood economic factors (poverty and income).

To strengthen the Discussion, the authors should explore potential contextual or environmental mechanisms that explain this specific association. For example, could this be driven by healthcare system strain in these neighborhoods (e.g., lower accessibility or quality of post-discharge care affecting all residents)? Could it be related to diminished social capital or increased social isolation for Swedish-born individuals living as a demographic minority in these areas? Alternatively, could it reflect residual confounding from the physical environment or higher community-level transmission of infectious diseases (including COVID-19)?

2.

It is plausible that limited spatial accessibility to medical facilities contributes to the higher mortality observed in these neighborhoods. Are there granular geographic data (e.g., postal

codes) available in the database that would allow for an analysis of this factor? If so, I recommend conducting a supplementary analysis to investigate whether the distance to healthcare explains this association.

3.

Regarding the discrepancy in outcomes between Western and non-Western migrants, the authors' explanation relying on "strong social bonds" (Page 10, Line 13) requires further justification.

If social support networks are truly protective, why wouldn't this apply to Western migrants as well? Moreover, a true protective effect should theoretically yield an IRR significantly below 1. Alternatively, could the non-significant association among non-Western migrants simply be the result of an insufficient sample size (underpowered analysis)? Please expand the Discussion to address these possibilities.

4.

Patients hospitalized with cancer represent a highly heterogeneous population with a wide range of reasons for admission and background comorbidities. Treating this broad demographic as a single, uniform cohort may oversimplify the analysis and introduce residual confounding.

Could the authors clarify whether their database contains more granular clinical information? Specifically, variables such as the primary diagnosis for hospitalization (e.g., cancer progression, infection, or other acute conditions), history of cancer-related surgeries, specific medications used (especially chemotherapy), level of consciousness at admission, or the level of care required (e.g., ICU, High Dependency Unit, or general ward) would be highly informative.

Incorporating these clinical details into the adjustment models, or conducting subgroup analyses based on these factors, would better account for baseline severity and add significant depth to the discussion.

minor comments:

1. Please clarify the data source used to obtain the data on disposable income.

2. Please correct the line numbering formatting before the next revision:

- The line numbers are misaligned with the text because blank lines are being counted.

- The numbering restarts on each page. Please change this to continuous line numbering throughout the manuscript.

VERSION 1 - AUTHOR RESPONSE

Reviewer: 1
Prof. Zoe Kelson, University of Exeter

Comments to the Author:
This retrospective cohort study examined whether neighbourhood migrant density influenced hospitalized cancer patients' outcomes, such as mortality and readmission rates, and whether these associations varied before and during the COVID-19 pandemic.
Response: Thank you for reviewing our manuscript. Please see below for our point-by-point responses.

Reviewer comments:

1. "Hospitalized cancer patients (ICD code C00-C97) from 2014-2019 (before the pandemic) and 2020-2021 (during the pandemic)" and "used multiple longitudinal national registers linked with unique pseudonymized personal identifiers, covering the total population of Sweden. The linked datasets include the total population register, the Longitudinal Integration Database for Health Insurance and Labour Market Studies (LISA), and the National Outpatient and Inpatient Register. " Can the authors please further comment on the completeness and coverage of this linked data?
Response: The total population register covers the entire population of Sweden from 1968 onwards. The Longitudinal Integration Database for Health Insurance and Labour Market Studies (LISA) contain information on all Swedish residents aged 16 years and above. Both the national outpatient and inpatient registers have nationwide coverage from both public and private healthcare providers. Therefore, using these nationwide registers allows complete coverage of the Swedish resident population. We have also included the information in the manuscript as well.

New Text: "The total population register covers the entire population of Sweden from 1968 onwards [21]. The Longitudinal Integration Database for Health Insurance and Labour Market Studies (LISA) contain information on all Swedish residents aged 16 years and above [22]. Both the national outpatient and inpatient registers have nationwide coverage from both public and private healthcare providers [23]. Therefore, using these nationwide registers allows complete coverage of the Swedish resident population.
We included individuals who were admitted to hospitals with cancer diagnosis. The study period was from 2014 to 2019 for "before the pandemic" period and from 2020 to 2021 for "during the pandemic" period." (Lines 99-107, Page 5)

2. "Individuals who had been hospitalized for cancer before the pandemic and survived were eligible to be included in the study during the pandemic period." Can the authors please conduct a sensitivity analysis on this, only comparing first hospitalisation outcomes for all individuals for example?
Response: Thank you for your suggestion. Our aim is to study the 90-day readmission and 90-day mortality following a hospitalization of cancer patients before and during the pandemic period. If we restrict to the first hospitalization, we will not be able to estimate the readmission outcome. Moreover, we will fail to capture the clinical course of cancer patients, who may experience multiple hospitalizations and reduce the substantial number of eligible hospitalizations, reducing the analytical power. Below are the number of readmissions,
Before Covid (2014 – 2019): 43.23 % (n=114.587) were readmitted
During Covid (2020 – 2022): 36.54 % (n=47.471) were readmitted.

3. "Conducted a complete case analysis, excluding individuals with missing data on outcomes,

exposures, or covariates as the missing percentage is low (less than 3 percent)." Can the authors please assess whether missing data can be assumed to be missing at random?
Response: Thank you for your comment. Upon revisiting the data, we identified that the previously reported missingness referred to the exposure dataset prior to linkage. These missing values in income, DeSO, and education were primarily due to individuals not residing in Sweden during the relevant period or having completed education abroad. However, after linking the exposure data with the hospitalized cancer patient dataset (i.e., the analytic sample used in this study), we found that there were no missing values in the variables included in the analysis. Therefore, a complete case analysis was conducted without missingness. We have corrected this in the revised manuscript.

New Text: "There were no missing values in the variables of the analytic dataset." (Lines 110, Page 6)

4. "Individual-level covariates included age (categorized into 5-year intervals), sex (male or female), education level (primary, secondary, or post-secondary), civil status (with or without a partner), the comorbidity index measured in the first year of observation, and the mean disposable income during the study period…Area-level deprivation for each DeSO unit was included as a time-fixed contextual covariate, measured at the first year of observation" Can the authors please include BMI in the study? Is data on lifestyle factors, such as smoking, alcohol, physical activity, available for inclusion?
Response: Thank you for your suggestions. National Swedish registry does unfortunately not include data on individual health behaviours or BMI since the data is linked from official administrative registers. We have added a note about this limitation.

New Text: "Finally, since we used the national registry data, we have no information on the individual health behaviours or BMI which can be potential confounders for the associations." (Lines 282-283, Page 12)

Reviewer: 2
Prof. Sakineh Hajebrahimi, Iranian Evidence Based Medicine Centre of Excellence, Tabriz University of Medical Sciences, Tabriz, Iran.

Comments to the Author:

1. The manuscript should more explicitly frame "high migrant density" not just as a demographic measure but as a marker of systemic marginalization. It likely correlates with under-resourced primary care, linguistic barriers, lower health literacy, and poorer social determinants of health for all residents. This framing is crucial for interpreting why Swedish-born individuals in these areas are also adversely affected.
Response: Thank you for reviewing our manuscript. We agree that neighbourhoods with high migrant density reflect structural marginalization rather than demographic composition alone. We stated the lower number of primary healthcare in these areas, and we added the information on the lack of health promoting services in the neighbourhood. While we acknowledge that linguistic barriers and health literacy may influence healthcare access, we consider these primarily individual-level factors rather than contextual neighbourhood characteristics. Therefore, we did not include them as neighbourhood-level determinants in the introduction.

New Text: "For cancer patients, though timely and effective care is ensured once treatment begins [14], migrant-dense neighbourhoods tend to have fewer primary healthcare practices [15],

which serve as the primary entry point to the healthcare services and are crucial for early detection of cancer. Limited availability of health promoting services, together with broader adverse social determinants, may affect healthcare access for all residents of these neighbourhoods [16].” (Lines 73-77, Page 4)

2. (a) The discussion on mechanisms is good but can be sharper. For the pandemic period, consider structuring the discussion around a clear conceptual model:
Direct Pathway: Higher COVID-19 incidence in these neighbourhoods → disruption of cancer care, nosocomial infection risk for immunocompromised patients.
Indirect Pathway: Fear of infection, misinformation, strained healthcare capacity → delayed presentations and follow-up → sicker patients at admission and discharge.
Response: *Thank you for your comment. We have organized the discussion using the two pathways.*

New Text: “First, since COVID-19 infection and hospitalization rates were higher in neighbourhoods with high migrant density [39], scheduled appointments of the cancer patients were significantly disrupted in these neighbourhoods, thereby limiting their access to adequate treatment and rehabilitation care, leading to an increased need for readmission [40]. Second, heightened fear of contracting the virus or transmitting it to others while accessing healthcare might have contributed to delayed cancer diagnoses and treatment and higher mortality rates among migrants during the pandemic [41 42].” (Lines 265-270, Page 11-12)

2. (b) The discussion on the potential protective "ethnic density effect" is appropriate. However, to strengthen this, consider formally testing an interaction between individual migrant status (non-Western) and neighbourhood non-Western migrant density. Does the (null/moderate) effect of density differ for non-Western migrants versus Swedish-born individuals? This could provide statistical support for the hypothesized protective buffering.
Response: We have conducted the interaction analysis between individual migration status and neighbourhood migrant density. We found a significant interaction for mortality among non-Western migrants, supporting “the ethnic density effect.” We have added the methods and results as below.

New Text: “Methods: To confirm the differences in associations between neighbourhood migrant density and outcomes by population groups, we conducted an interaction analysis.” (Lines 165-167, Page 8)
“Results: Table 3 shows the associations between neighbourhood migrant density and the 90-day mortality following cancer hospitalization, before and during the pandemic, with p-values for the interactions between migrant density and population groups…. Associations between neighbourhood migrant density and the 90-day mortality among non-Western migrants were not statistically significant both before and during the pandemic. However, p for interaction for these migrants were significant (p=0.02).” (Lines 184-196, Page 8-9)
“Discussion: There were no significant associations between neighbourhood migrant density and mortality and readmission among non-Western migrants before the pandemic. However, we observed a significant interaction indicating that associations of migrant density differ
red between non-Western migrants and Swedish-born individuals, suggesting that the neighbourhood migrant density operates differently across population groups….
Notably, the previously observed interaction between migrant density and migrant status was no longer significant during the pandemic, showing the possible disruption of different associations across migrant groups.” (Lines 244-262, Page 11)

Table 3: Association between neighbourhood migrant density and the 90-day mortality among hospitalized cancer patients, before the pandemic and during the pandemic

	**Before the pandemic (2014-2019)**				**During the pandemic (2020-2021)**			
	Model 1	Model 2	P for interaction	Forest plot (Model 2)	Model 1	Model 2	P for interaction	Forest plot (Model 2)
**For Swedish-born**				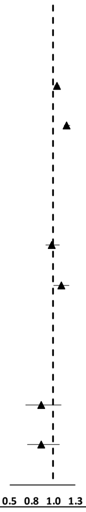				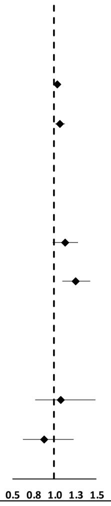
Low	ref	ref	ref		ref	ref	ref	
Medium	1.04 (1.02, 1.07) *	1.04 (1.01, 1.07) *	ref		1.03 (0.99, 1.08)	1.03 (0.99, 1.07)	ref	
High	1.15 (1.12, 1.19) *	1.15 (1.12, 1.19) *	ref		1.09 (1.05, 1.14) *	1.06 (1.01, 1.12) *	ref	
**For Western migrants**								
Low	ref	ref	ref		ref	ref	ref	
Medium	0.98 (0.91, 1.06)	0.98 (0.91, 1.07)	0.18		1.11 (0.97, 1.26)	1.12 (0.98, 1.28)	0.37	
High	1.07 (1.00, 1.16)	1.09 (1.00, 1.18) *	0.16		1.16 (1.02, 1.31) *	1.25 (1.09, 1.43) *	0.88	
**For non-Western migrants**								
Low	ref	ref	ref		ref	ref	ref	
Medium	0.89 (0.70, 1.12)	0.86 (0.68, 1.09)	0.07		1.05 (0.76, 1.46)	1.07 (0.77, 1.49)	0.61	
High	0.91 (0.75, 1.12)	0.86 (0.70, 1.07)	0.02*		1.07 (0.79, 1.44)	0.88 (0.63, 1.23)	0.82	

Model 1: Adjusted for age, sex, disposable income, education, civil status and comorbidity index.

Model 2: Model 1+ Area level deprivation index

Neighbourhood migrant density was calculated by dividing the population of each group in a specific geographic area by the total population of that area. * p-value <0.05

Table 4: Association between neighbourhood migrant density and the 90-day readmission among hospitalized cancer patients, before the pandemic and during the pandemic

	**Before the pandemic (2014-2019)**				**During the pandemic (2020-2021)**			
	Model 1	Model 2	P for interaction	Forest plot (Model 2)	Model 1	Model 2	P for interaction	Forest plot (Model 2)
**For Swedish-born**				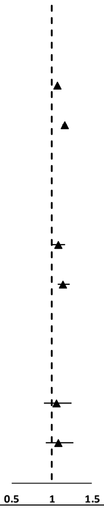				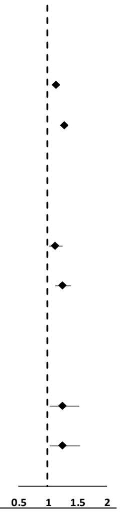
Low	ref	ref	ref		ref	ref	ref	
Medium	1.06 (1.04, 1.08) *	1.07 (1.05, 1.09) *	ref		1.11 (1.08, 1.15) *	1.14 (1.10, 1.18) *	ref	
High	1.13 (1.10, 1.16) *	1.16 (1.13, 1.19) *	ref		1.18 (1.14, 1.22) *	1.29 (1.23, 1.34) *	ref	
**For Western migrants**								
Low	ref	ref	ref		ref	ref	ref	
Medium	1.07 (1.00, 1.14)	1.08 (1.01, 1.16) *	1.00		1.11 (1.01, 1.23) *	1.13 (1.02, 1.25) *	0.82	
High	1.09 (1.03, 1.16) *	1.14 (1.07, 1.22) *	0.89		1.06 (0.97, 1.17)	1.25 (1.12, 1.40) *	0.40	
**For non-Western migrants**								
Low	ref	ref	ref		ref	ref	ref	
Medium	1.06 (0.90, 1.25)	1.06 (0.90, 1.25)	0.23		1.21 (0.99, 1.48)	1.26 (1.03, 1.54) *	0.06	
High	1.07 (0.92, 1.24)	1.09 (0.93, 1.27)	0.73		1.15 (0.96, 1.39)	1.26 (1.03, 1.55) *	0.72	

Model 1: Adjusted for age, sex, disposable income, education, civil status and comorbidity index.

Model 2: Model 1+ Area level deprivation index

Neighbourhood migrant density was calculated by dividing the population of each group in a specific geographic area by the total population of that area. * p-value<0.05

2. (c) Acknowledge that the younger age of non-Western migrants is a major competing explanation for their lower mortality. Could you run a sensitivity analysis age-standardizing the rates or presenting an age-stratified analysis (<65 vs. 65+)?

Response: We conducted an age-stratified analysis for individuals younger than 65 and those aged 65 and older. Overall, there were more significant associations for those above 65 years old. Before the pandemic, we found that the direction of associations for non-Western migrants

was similar for both age groups. This suggests the younger age of non-Western migrants is not a sole determining factors for their lower mortality.

New Text: "Methods: To account for the age distributions between Swedish-born and migrant populations, we conducted age-stratified analysis, categorising participants into age groups younger than 65 and 65 years and older for all outcomes, as a sensitivity analysis." (Lines 167-169, Page 8)
"Results: Supplementary Table 4-7 describes age-stratified associations between neighbourhood migrant density and the outcomes among individuals younger than 65 and those aged 65 years and older. Statistically significant associations for the mortality were primarily observed among individuals aged 65 years and older." (Lines 210-213, Page 9)

3. The analysis presents results for three exposure variables: total, Western, and non-Western migrant density. Which is considered the primary exposure? The rationale for analysing all three should be stated upfront in the methods. It might be clearer to present total migrant density as the main result in the abstract and core tables, with Western/non-Western breakdowns as stratified or sensitivity analyses.
Response: Thank you for your comment. We have added the rationale for analysing three exposures in the methods section. We also divided the results into primary exposure "migrant density" (Table 3-4) and stratified exposure "Western/non-Western migrant density" (Supplementary Table 2-3).

New Text: "The main exposure, migrant density, a compositional measure of migrant residential segregation [26], was calculated by dividing the migrant population in a specific geographic area by the total population of that area. We then stratified this measure into Western and non-Western migrant density, as supplementary analyses, to examine whether associations differed across migrant groups residing in neighbourhoods with similar or different migrant background compositions." (Lines 125-129, Page 6)

4. Consider reporting measures of model fit (e.g., Variance Partition Coefficient - VPC) from your multilevel models. How much of the total variance in outcomes is explained at the neighbourhood (DeSO) level? Does adding migrant density and area deprivation substantially reduce this between-area variance?
Response: Thank you for your comment. We calculated interclass correlation coefficient (ICC), equivalent to the VPC, of the multilevel models.
ICCs decreased after adjustment of area deprivation, suggesting that this factor explains part of the between-area variance. Overall, ICCs were small across models, indicating limited area variation. Despite this, we retained the multilevel modelling approach to account for the hierarchical data structure, as even small ICCs may lead to inflate Type 1 error rates if clustering is ignored (Musca et al., 2011). The Methods section has been revised accordingly.

New text: "Model fit in the multilevel analyses was assessed using the intraclass correlation coefficient (ICC), which ranges up to 0.4. Despite small ICC values, we maintained multilevel models to account for the hierarchical structure of data, as ignoring clustering may result in underestimated standard errors, even with low between-group variance [29]." (Lines 157-160, Page 7)

5. Forest plots showing IRRs and 95% CIs for "High vs. Low" migrant density for mortality and readmission, stratified by population group (Swedish/Western/Non-Western) and period

(Pre/During Pandemic). This would allow readers to instantly grasp the magnitude, precision, and patterns of the main effects.

Response: Thank you for your comment. We prepared forest plots for the associations.

New Text:

Table 3: Association between neighbourhood migrant density and the 90-day mortality among hospitalized cancer patients, before the pandemic and during the pandemic

	**Before the pandemic (2014-2019)**				**During the pandemic (2020-2021)**			
	Model 1	Model 2	P for interaction	Forest plot (Model 2)	Model 1	Model 2	P for interaction	Forest plot (Model 2)
**For Swedish-born**				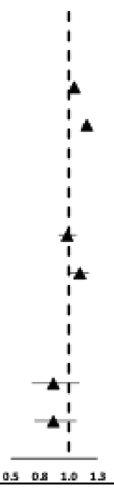				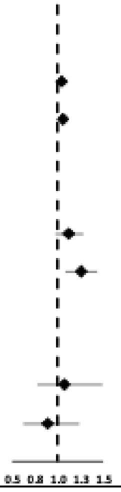
Low	ref	ref	ref		ref	ref	ref	
Medium	1.04 (1.02, 1.07) *	1.04 (1.01, 1.07) *	ref		1.03 (0.99, 1.08)	1.03 (0.99, 1.07)	ref	
High	1.15 (1.12, 1.19) *	1.15 (1.12, 1.19) *	ref		1.09 (1.05, 1.14) *	1.06 (1.01, 1.12) *	ref	
**For Western migrants**								
Low	ref	ref	ref		ref	ref	ref	
Medium	0.98 (0.91, 1.06)	0.98 (0.91, 1.07)	0.18		1.11 (0.97, 1.26)	1.12 (0.98, 1.28)	0.82	
High	1.07 (1.00, 1.16)	1.09 (1.00, 1.18) *	0.16		1.16 (1.02, 1.31) *	1.25 (1.09, 1.43) *	0.4	
**For non-Western migrants**								
Low	ref	ref	ref		ref	ref	ref	
Medium	0.89 (0.70, 1.12)	0.86 (0.68, 1.09)	0.07		1.05 (0.76, 1.46)	1.07 (0.77, 1.49)	0.06	
High	0.91 (0.75, 1.12)	0.86 (0.70, 1.07)	0.02*		1.07 (0.79, 1.44)	0.88 (0.63, 1.23)	0.72	

Model 1: Adjusted for age, sex, disposable income, education, civil status and comorbidity index.

Model 2: Model 1+ Area level deprivation index

Neighbourhood migrant density was calculated by dividing the population of each group in a specific geographic area by the total population of that area.
* p-value<0.05

Table 4: Association between neighbourhood migrant density and the 90-day readmission among hospitalized cancer patients, before the pandemic and during the pandemic

	**Before the pandemic (2014-2019)**				**During the pandemic (2020-2021)**			
	Model 1	Model 2	P for interaction	Forest plot (Model 2)	Model 1	Model 2	P for interaction	Forest plot (Model 2)
**For Swedish-born**				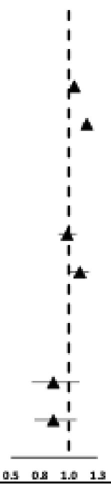				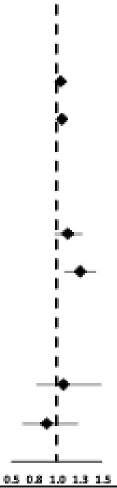
Low	ref	ref	ref		ref	ref	ref	
Medium	1.04 (1.02, 1.07) *	1.04 (1.01, 1.07) *	ref		1.03 (0.99, 1.08)	1.03 (0.99, 1.07)	ref	
High	1.15 (1.12, 1.19) *	1.15 (1.12, 1.19) *	ref		1.09 (1.05, 1.14) *	1.06 (1.01, 1.12) *	ref	
**For Western migrants**								
Low	ref	ref	ref		ref	ref	ref	
Medium	0.98 (0.91, 1.06)	0.98 (0.91, 1.07)	0.18		1.11 (0.97, 1.26)	1.12 (0.98, 1.28)	0.82	
High	1.07 (1.00, 1.16)	1.09 (1.00, 1.18) *	0.16		1.16 (1.02, 1.31) *	1.25 (1.09, 1.43) *	0.4	
**For non-Western migrants**								
Low	ref	ref	ref		ref	ref	ref	
Medium	0.89 (0.70, 1.12)	0.86 (0.68, 1.09)	0.07		1.05 (0.76, 1.46)	1.07 (0.77, 1.49)	0.06	
High	0.91 (0.75, 1.12)	0.86 (0.70, 1.07)	0.02*		1.07 (0.79, 1.44)	0.88 (0.63, 1.23)	0.72	

Model 1: Adjusted for age, sex, disposable income, education, civil status and comorbidity index.

Model 2: Model 1+ Area level deprivation index

Neighbourhood migrant density was calculated by dividing the population of each group in a specific geographic area by the total population of that area.
* p-value<0.05

6. The discussion should more forcefully propose testable mechanisms for the observed disparities. For example, are there data on the density of oncology clinics, palliative care

services, or primary care physicians with cancer expertise across DeSO areas? Even citing related literature would help.
Response: Thank you for your comment. Unfortunately, we have no data on the disparities of oncology care across DeSO areas. However, we included literature on the regional disparities of the palliative care and specialist cancer therapy in Sweden.

New Text: "Although Sweden provides universally accessible, high-quality care, regional disparities for the provision of palliative care and specialist therapy, were present, particularly in socioeconomically disadvantaged areas [30 31]." (Lines 229-231, Page 10)

7. The conclusion is good but can be more specific and actionable. What does "improve cancer care" mean in practice?
Recommendation 1: Targeted resource allocation for hospital discharge planning and community-based support in high-density neighbourhoods.
Recommendation 2: Developing pandemic preparedness plans that explicitly address the vulnerability of cancer patients in marginalized neighbourhoods (e.g., protected cancer care pathways, community outreach to address fear).
Recommendation 3: Investment in community health workers and interpreters embedded within these neighbourhoods to bridge hospital and home care. Briefly contextualize the findings beyond Sweden. While Sweden's universal system is unique, the clustering of marginalized populations in under-resourced urban areas is a global phenomenon. The lessons about how such area-level vulnerability interacts with a health system shock are universally important.
Response: Thank you for your detailed comment. We agree that community-based support is essential for the vulnerable population in the deprived neighbourhoods. We have updated the conclusion section as below.

New Text: "Cancer care resources should be allocated to neighbourhoods with high migrant density to invest in community health workers and interpreters, as well as in improving community-based support, to promote after-discharge care and bridge the gap between hospital and home care. During crisis situations, pandemic preparedness plans are necessary to address vulnerable populations in marginalised neighbourhoods. These plans should include developing cancer care pathways and community outreach programmes to address the fear of the pandemic, and they could be adapted globally." (Lines 291-297, Page 12-13)

Minor Revisions

1. Abstract Results: The abstract states mortality rates "slightly increased among Western migrants" during the pandemic. The result (IRR: 1.22, 95% CI: 1.07, 1.39) suggests a 22% increase, which is substantial. Consider rephrasing to "significantly increased."
Response: Thank you for your comment. We have changed the wordings as suggested.

2. Missing Data: The statement "missing percentage is low (less than 3 percent)" is reassuring. Specify if this is for all variables combined or for key variables. A supplemental table showing patterns of missingness would be ideal.
Response: Thank you for your comment. Upon revisiting the data, we identified that the previously reported missingness referred to the exposure dataset prior to linkage. These missing values in income, DeSO, and education were primarily due to individuals not residing in Sweden during the relevant period or having completed education abroad. However, after linking the exposure data with the hospitalized cancer patient dataset (i.e., the analytic sample

used in this study), we found that there were no missing values in the variables included in the analysis. Therefore, a complete case analysis was conducted without missingness. We have corrected this in the revised manuscript.

New Text: "There were no missing values in the variables of the analytic dataset." (Lines 110, Page 6)

Reviewer: 3
Dr. Takuya Sato, The University of Tokyo Hospital
Comments to the Author:

Dear Authors,
Thank you for the opportunity to review your manuscript "Neighbourhood migrant density and outcomes in hospitalized cancer patients before and during the COVID-19 pandemic in Sweden: A register-based study". This retrospective observational cohort study investigated the association between neighbourhood migrant density and the mortality after cancer hospitalization. The results demonstrated a significantly higher mortality rate among the Swedish-born population and western immigrants living in high migrant density area. The perspective is novel and interesting. However, I have a few comments and questions that may help clarify the findings for the readers.
Response: Thank you for reviewing our manuscript. Please see below for our point-by-point responses.

1. The authors state, *"This discrepancy may be because our study focused on the mortality among hospitalized cancer patients, who are particularly vulnerable for factors beyond economic deprivation."* (Page 9, Line 33)
While I agree that cancer patients are highly vulnerable, this statement only explains an individual-level susceptibility, not the neighbourhood-level mechanism. It does not explain *why* living in a high-migrant-density area exacerbates this vulnerability even after adjusting for individual and neighbourhood economic factors (poverty and income).
To strengthen the Discussion, the authors should explore potential contextual or environmental mechanisms that explain this specific association. For example, could this be driven by healthcare system strain in these neighbourhoods (e.g., lower accessibility or quality of post-discharge care affecting all residents)? Could it be related to diminished social capital or increased social isolation for Swedish-born individuals living as a demographic minority in these areas? Alternatively, could it reflect residual confounding from the physical environment or higher community-level transmission of infectious diseases (including COVID-19)?
Response: Thank you for your comment. We agree that the vulnerability of cancer patients living in these areas may be due to factors beyond individual circumstances. We found evidence of regional disparities in oncology specialist care, which may be particularly relevant for our population. We included this fact in the discussion section. We also included about higher COVID-19 risk among immunocompromised cancer patients. However, to the best of our knowledge, we could not find literature on the social isolation of Swedish-born individuals living in migrant-dense neighbourhoods.

New Text: "Although Sweden provides universally accessible, high-quality care, regional disparities for the provision of palliative care and specialist therapy, were present, particularly in socioeconomically disadvantaged areas [30 31]." (Lines 229-231, Page 10)
"First, since COVID-19 infection and hospitalization rates were higher in neighbourhoods with high migrant density [39], scheduled appointments of the cancer patients were significantly

disrupted in these neighbourhoods, thereby limiting their access to adequate treatment and rehabilitation care, leading to an increased need for readmission [40].” (Lines 265-268, Page 11)

2. It is plausible that limited spatial accessibility to medical facilities contributes to the higher mortality observed in these neighbourhoods. Are there granular geographic data (e.g., postal codes) available in the database that would allow for an analysis of this factor? If so, I recommend conducting a supplementary analysis to investigate whether the distance to healthcare explains this association.
Response: Thank you for your comment. Unfortunately, we do not have postal codes data for the participants that we could not examine the potential explanation of the distance to healthcare.

3. Regarding the discrepancy in outcomes between Western and non-Western migrants, the authors' explanation relying on "strong social bonds" (Page 10, Line 13) requires further justification.
If social support networks are truly protective, why wouldn't this apply to Western migrants as well? Moreover, a true protective effect should theoretically yield an IRR significantly below 1. Alternatively, could the non-significant association among non-Western migrants simply be the result of an insufficient sample size (underpowered analysis)? Please expand the Discussion to address these possibilities.
Response: Thank you for your comment. Due to the smaller sample size of non-Western migrants, the confidence intervals are wider, and the associations did not reach statistical significance for both positive and negative associations. However, it is noteworthy that the point estimates for mortality were consistently lower in neighbourhoods with higher migrant density compared to those with lower density, which may suggest a modest protective association.
We formally tested an interaction between migrant type and neighbourhood migrant density. The results indicated a positive interaction for non-Western migrants, suggesting that the association between neighbourhood composition and outcomes differ by migrant group, which is also cited in the previous literature (Cederström, A., 2025). Nevertheless, we agreed that the register data do not include information on social ties, so we cannot conclude why these strong social bonds are not protective for the Western migrants.

4. Patients hospitalized with cancer represent a highly heterogeneous population with a wide range of reasons for admission and background comorbidities. Treating this broad demographic as a single, uniform cohort may oversimplify the analysis and introduce residual confounding. Could the authors clarify whether their database contains more granular clinical information? Specifically, variables such as the primary diagnosis for hospitalization (e.g., cancer progression, infection, or other acute conditions), history of cancer-related surgeries, specific medications used (especially chemotherapy), level of consciousness at admission, or the level of care required (e.g., ICU, High Dependency Unit, or general ward) would be highly informative. Incorporating these clinical details into the adjustment models, or conducting subgroup analyses based on these factors, would better account for baseline severity and add significant depth to the discussion.
Response: Thank you for your comment. We agree that adding additional information on hospitalization would enrich the discussion. However, this is a major limitation of this study, as we only have the date of hospitalization and the main diagnosis at the time of hospitalization, which is cancer or not. We have also included this information to the limitations.

New Text: "Third, we were unable to account for detailed diagnosis and treatment types received by patients, which are the important determinants of the mortality and readmission of hospitalized cancer patients, as this information was not available in our data." (Lines 276-279, Page 12)

Minor comments:

1. Please clarify the data source used to obtain the data on disposable income.
Response: We have added the data source for the disposable income.

New Text: "The income information was derived from the LISA database, which collects income data from national registers including those of the Swedish tax authorities and social welfare services [22]." (Lines 144-146, Page 7)

2. Please correct the line numbering formatting before the next revision:
-The line numbers are misaligned with the text because blank lines are being counted.
-The numbering restarts on each page. Please change this to continuous line numbering throughout the manuscript.
Response: Thank you for your comment. In the previous version, line numbers are automatically added by the submission system, so we added line numbers by ourselves this time.

VERSION 2 - REVIEW

**Reviewer**	**1**
**Name**	**Kelson, Zoe**
**Affiliation**	**University of Exeter, Mathematics**
**Date**	**15-Apr-2026**
**COI**	

Thanks to the authors for their responses, clarifications, and revisions.
